# HOXB7 overexpression promotes cell proliferation and correlates with poor prognosis in gastric cancer patients by inducing expression of both AKT and MARKs

**DOI:** 10.18632/oncotarget.13604

**Published:** 2016-11-25

**Authors:** Xujun He, Zhengchuang Liu, Yingjie Xia, Ji Xu, Guocai Lv, Lu Wang, Tonghui Ma, Liping Jiang, Yiping Mou, Xiaoting Jiang, Jie Ma, Zhongkuo Zhao, Haibin Ni, Wenjuan Xu, Guoqing Ru, Dongsheng Huang, Houquan Tao

**Affiliations:** ^1^ Key Laboratory of Gastroenterology of Zhejiang Province, Hangzhou, 310014, China; ^2^ Department of Surgery, Zhejiang Provincial People's Hospital, Hangzhou 310014, Zhejiang, China; ^3^ Department of Laboratory Medicine, First Affiliated Hospital, College of Medicine, Zhejiang University, Hangzhou 310003, China; ^4^ Key Laboratory of Clinical *In vitro* Diagnostic Techniques of Zhejiang Province, Hangzhou 310003, China; ^5^ Department of Pathology, Zhejiang Provincial People's Hospital, Hangzhou 310014, Zhejiang, China; ^6^ Department of Cardiothoracic Surgery, Zhejiang Provincial People's Hospital, Hangzhou 310014, Zhejiang, China; ^7^ Department of Surgery, Tongde Hospital of Zhejiang Province, Hangzhou 310012, Zhejiang, China

**Keywords:** HOXB7, gastric cancer, prognosis, AKT, MAPKs

## Abstract

Increased expression of *HOXB7* has been reported to correlate with the progression in many cancers. However, the specific mechanism by which it promotes the evolution of gastric cancer (GC) is poorly understood.

In this study, we sought to investigate the role of *HOXB7 in GC* by assessing *HOXB7* expression in patient tissue and its correlation to clinical characteristics. We found that GC tissues showed increased expression of *HOXB7* and that the *HOXB7* expression was significantly associated with Lauren classification, invasion depth, lymphatic metastasis and poor prognosis, and could serve as an independent prognostic factor. To further investigate the role of HOXB7 in GC, we generated stable GC cell lines and both over-expressed and knocked down *HOXB7* expression. Over-expression of *HOXB7 in GC cell* lines enhanced cell proliferation, colony formation, migration and invasion ability, whereas the opposite trends were observed upon reduction of *HOXB7* expression by knockdown. These findings were further supported by our *in vivo* studies which show that *HOXB7* expression can affect the GC cells' subcutaneous growth and lung metastases. A Phospho-MAPK Array Kit was used to explore the possible mechanism of HOXB7-induced cell proliferation and invasion. We found that the AKT signaling pathway and the two members of the MAPK pathway, were involved in those promoting effects. In conclusion, our results showed that increased expression of *HOXB7* might play an important role in promoting GC proliferation, migration and invasion by inducing both AKT and MAPK pathways, thus resulting in progression of, and poor prognosis in GC patients.

## INTRODUCTION

Despite improvements in surgical technique and adjuvant chemotherapy, gastric cancer (GC) remains a highly lethal disease. In China, the five year survival rate of GC is only 40% and GC ranks as the second most frequent cause of cancer-related deaths [1–2]. Unfortunately, GC is often diagnosed at advanced stages (III-IV), when metastatic disease with lymphatic spread is common. Better understanding the molecular mechanisms of GC is urgently needed to identify novel therapeutic targets and identify biomarkers for GC prognosis.

The homeobox genes encode a family of transcription factors that are essential for regulating growth and differentiation during embryonic development and maintaining adult tissue homeostasis. They are frequently dysregulated in cancer where they variably impact tumor cells proliferation, migration, invasion, apoptosis [3–4]. Thirty-nine HOX genes were categorized into four chromosomal clusters (A, B, C and D) have been reported in human. These four chromosomal clusters, each about 100 kb in length, are located on chromosomes 7, 17, 2, and 12, respectively [5]. As a member of class I homeobox genes, the transcription factor homeobox B7 (*HOXB7*) is known to play an important role in tumorigenesis in several cancers, including melanoma [6], breast [7], lung [8], colorectal [9] and pancreatic cancers [10]. Overexpression of *HOXB7* is correlated with poor prognosis of patients with many different cancers, such as esophageal squamous cell carcinoma [11–13], colorectal cancer [9] and oral cancer [14]. Studies have shown that in these cancers, *HOXB7* overexpression promotes cell proliferation, DNA repair [15], angiogenesis, epithelial mesenchymal transition (EMT) and cell survival [16]. Recently, it was reported that HOXB7 plays a dual role in HER2 positive breast cancer progression by delaying tumor formation, but promoting lung metastasis [17]. Although many studies have shown that HOXB7 plays an important role in cancer development, the biological functions of HOXB7 in GC tumorigenesis, progression and prognosis have not been well characterized. Here, we aimed to investigate the prognostic significance and possible functional mechanisms of HOXB7 in GC.

## RESULTS

### *HOXB7* was up-regulated in GC tissues and cell lines

**Figure 1:**
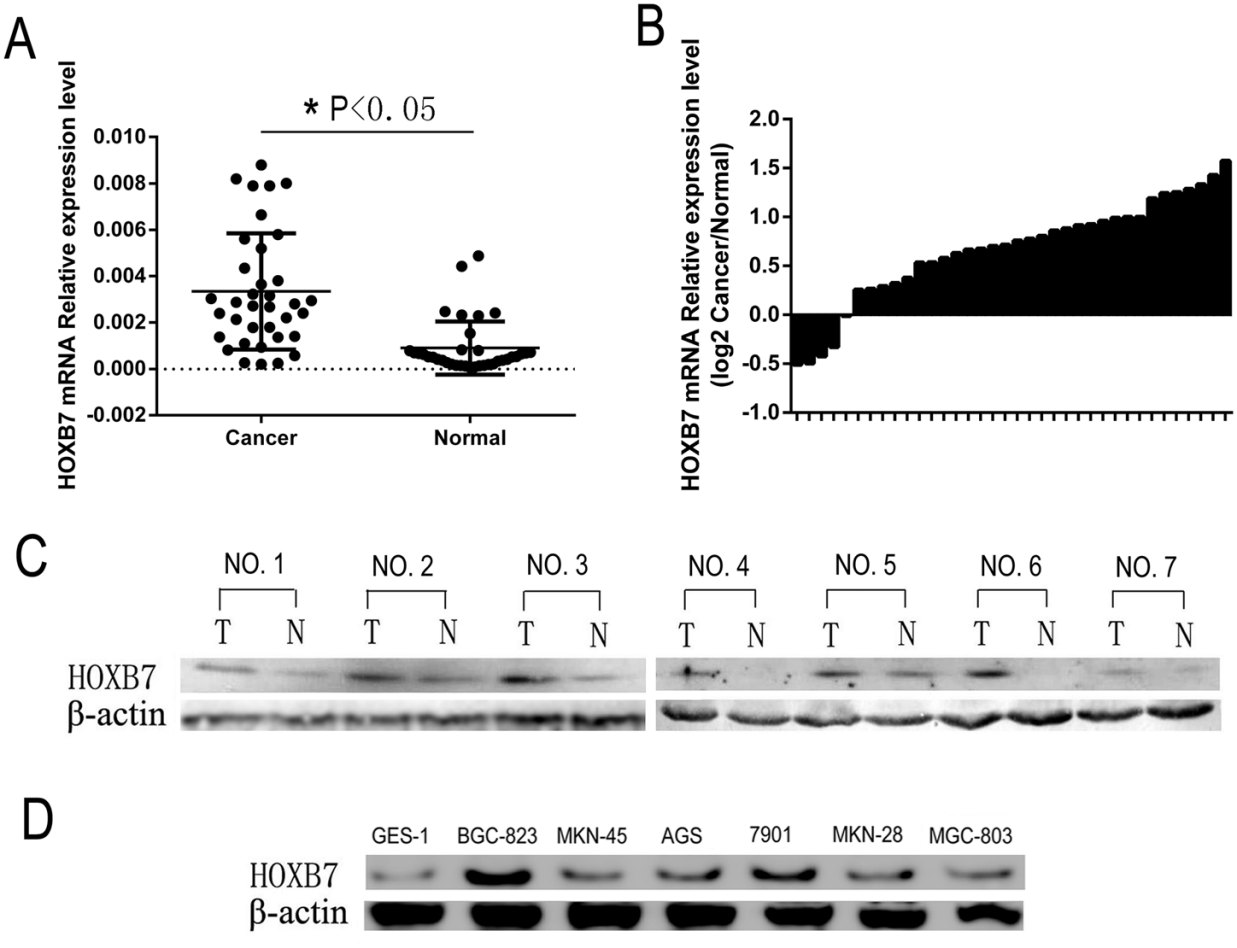
HOXB7 expression in GC samples and cell lines **A.** and **B.** *HOXB7* mRNA expression levels were measured in 36 paired (tumor and adjacent normal tissue) GC tissues by real-time PCR relative to *GAPDH* expression. A, *HOXB7* mRNA expression levels are increased in GC tumor tissue relative to adjacent normal tissues (Cancer *vs.* Normal, *P*<0.05). B, *HOXB7* mRNA expression level in 36 paired GC tissues (Log2 Cancer *vs.* Normal). **C.** Expression of HOXB7 protein in primary GC tissues (T) and the paired tissues adjacent noncancerous tissues (N) from the same patient by western blotting, and in each of the T and N tissues β-Actin was used as an internal control. **D.** The protein expression level of HOXB7 in GC cell lines. Expression levels were normalized with β-Actin.

Expression of *HOXB7* was analyzed by qPCR in 36 GC patients' tissues, in both tumor and paired adjacent noncancerous regions of the tissues. Comparative analysis indicated that expression of *HOXB7* was significantly increased in GC tumor tissue relative to adjacent noncancerous gastric tissues (*P*<0.05, Figure 1A). Only 11% (4/36) of GC patient tissues showed lower *HOXB7* expression in cancer tissues relative to paired adjacent noncancerous tissue (Figure 1B). The expression levels of HOXB7 were much higher in GC (0.003344 ±0.0004176) tissues than in noncancerous tissues (0.0009040 ±0.0001908; *P*<0.01; Figure 1A). Furthermore, increased levels of HOXB7 protein was found in GC tissue relative to adjacent normal tissue levels in seven GC patient cases by Western blot (Figure 1C). Additionally, Western blot analysis of six GC cell lines, including BGC-823, MKN-45, 7901, AGS, MKN-28 and MGC-803, exhibited increased levels of HOXB7 in comparison to the normal gastric mucosa cell line GES-1, and among the six GC lines, the HOXB7 expression was the lowest in MGC-803 and the highest in BGC-823 (Figure 1D).

### *HOXB7* expression promotes proliferation and invasiveness of GC cell lines *in vitro*

**Figure 2:**
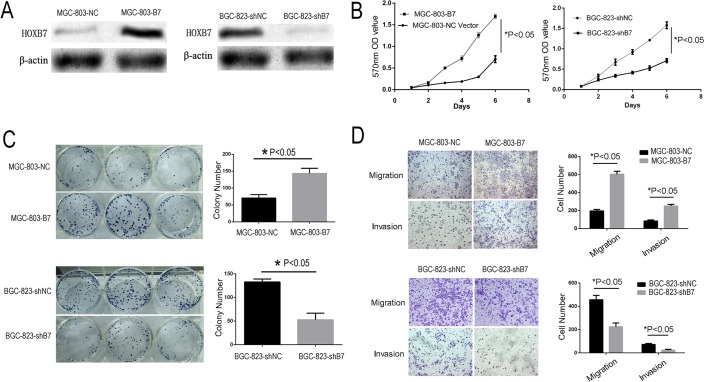
Increased HOXB7 expression promotes gastric cancer cell line proliferation, clone formation, migration and invasion **A.** The expression level of HOXB7 change in MGC-803 and BGC-823 cells, which stable over and known-down HOXB7 express, respectively. **B.** Dysregulated expression of HOXB7 in MGC-803 and BGC-823 cells affect cell proliferation determined by MTT assays. **C.** Colony formation assays **D.** Cell migration and invasion assays. Conclusion, Up-regulation the HOXB7 express could stimulate GC cell proliferation, clone formation, migration and invasion, whereas knock-down of HOXB7 expression GC cell could inhibit these effects. Error bars represent Mean ±SD from 3 independent experiments. *, *P* < 0.05.

To evaluate the possible role of HOXB7 in the tumorigenesis and invasiveness of human GC cells, we generated a *HOXB7* shRNA knockdown GC cell line BGC-823-shB7, and a *HOXB7* overexpression GC cell line, MGC-803-B7 (Figure 2A). MGC-803 and BGC-823 were selected for further study because these two GC cell lines were found to have relatively lower and higher endogenous HOXB7 expression, respectively, than other GC cell lines (Figure 1D).

Overexpression of *HOXB7 in the GC line* MGC-803 resulted in significantly increased proliferation relative to the negative control cell line (MGC-803-NC) by the MTT assay (Figure 2B; *P*<0.05). The colony formation assay revealed that these MGC-803-B7 also cells formed significantly more and larger colonies than control cells (Figure 2C; *P*<0.01). The MGC-803 lines also showed significantly increased migration and invasive ability with overexpression of *HOXB7* relative to control the control line by the Transwell assay (Figure 2D; *P* <0.05). In order to further validate the phenotypic differences we saw upon expression of *HOXB7* expression in GC cell lines, we knocked down endogenous HOXB7 in BGC-823, which normally expresses elevated levels of *HOXB7*, by shRNA-mediated knockdown (Figure 2A). Knockdown of *HOXB7 in BGC-823 resulted in significantly decreased proliferation rate*, decreased colony formation ability, and decreased migration and invasion by the MTT assay, colony formation assay, migration and invasion assays, respectively (Figure 2B–2D; *P*<0.05).

### *HOXB7* expression promotes tumorigenesis and invasiveness of GC *in vivo*

The *in vitro* data indicated that the expression of HOXB7 conferred features of tumorigenesis and invasiveness to GC cell lines. To test whether expression of HOXB7 could promote these features of GC *in vivo*, MGC-803-B7 and BGC-823-shB7 and their respective controls (MGC-803-NC and BGC-823-shNC) cell lines were studied both through subcutaneous injection and also by tail vein injection in immunodeficient female nude mice.

**Figure 3:**
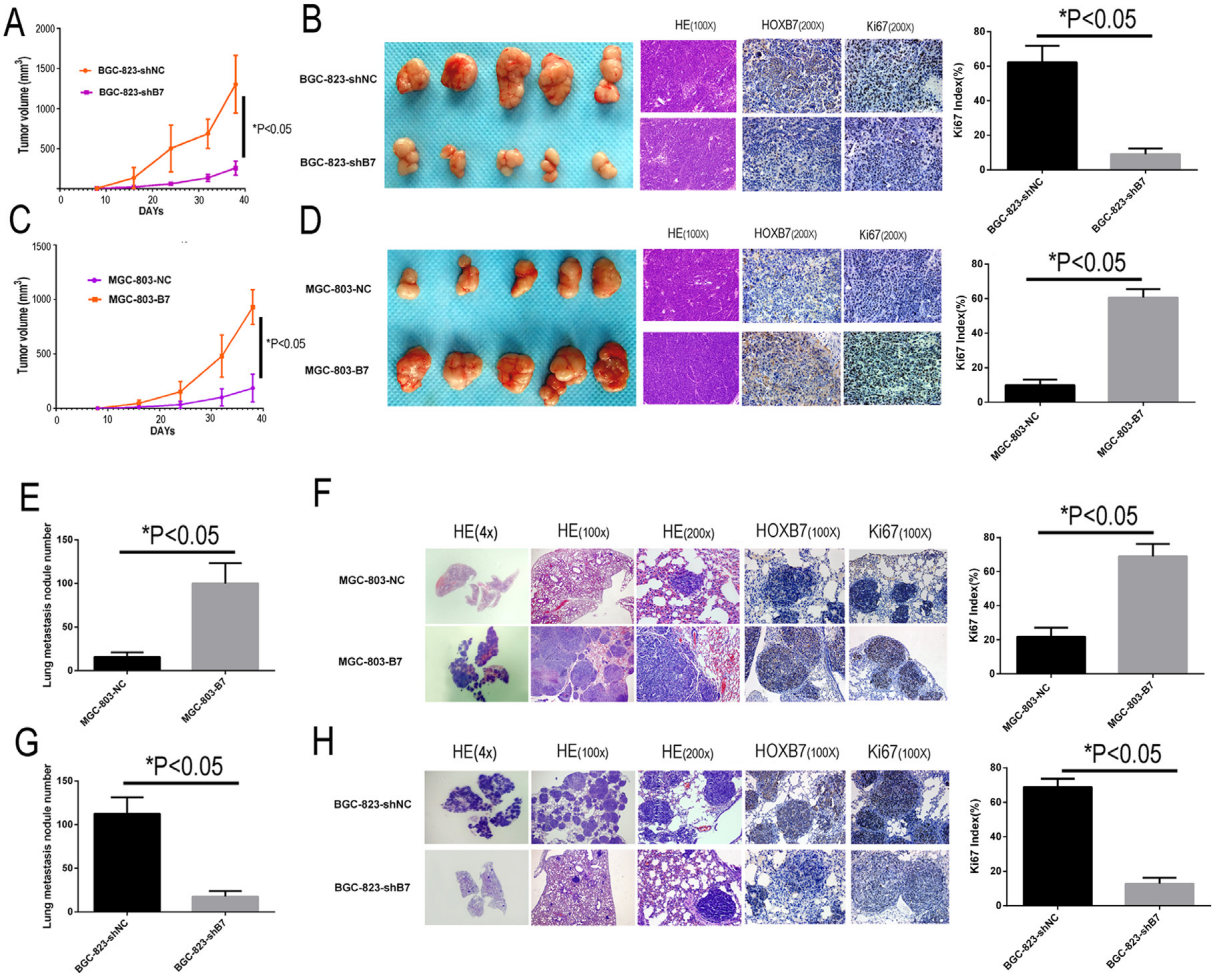
HOXB7 expression promotes tumorigenesis and lung metastasis in subcutaneous implantation and tail vein injection models Up-regulation the HOXB7 express could stimulate GC cell subcutaneous tumor growth and lung metastasis in nude mice. IHC stain indicated that up-regulation the HOXB7 express the tumor sections had high HOXB7 and Ki-67 immune staining intensity, both in subcutaneous tumor sections and lung metastasis sections, whereas knock-down the HOXB7 express could get the opposite result, compared with each negative control group. **A.** Silencing endogenous HOXB7 inhibited cell growth of BGC-823 in nude mice subcutaneously transplanted model. Data points are presented as the mean tumor Volume ± SD. **B.** General picture of the subcutaneous tumor block of BGC-823-shNC and BGC-823-shB7 group. The tumor sections were under H&E staining and IHC staining for HOXB7 and Ki-67. **C.** Over-expression of HOXB7 promotes cell growth of MGC-803 in nude mice in a subcutaneously transplanted model. Data points are presented as the mean tumor Volume ± SD. **D.** General picture of the subcutaneous tumor block of MGC-803-NC and MGC-803-B7 group. The tumor sections were under H&E staining and IHC staining for HOXB7 and Ki-67. **E.** The lung metastasis nodule number of MGC-803-NC and MGC-803-B7 group. **F.** Different magnification picture of lung metastasis nodule of BGC-823-shNC and BGC-823-shB7 group under H&E staining and IHC staining result of HOXB7 and Ki-67. **G.** The lung metastasis nodule number of BGC-823-shNC and BGC-823-shB7 group. **H.** Different magnification picture of lung metastasis nodule of BGC-823-shNC and BGC-823-shB7 group under H&E staining and IHC staining result of HOXB7 and Ki-67. Error bars represent Mean ±SD from 3 independent experiments. *, *P* < 0.05.

In subcutaneous implantation models, shRNA-mediated reduction in *HOXB7* expression in the BGC-823 cell line caused significant reduction tumor growth relative to controls (1301.38 ± 294.25 mm^3^ in BGC-823-shNC group *vs.* 255.79 ± 72.35 mm^3^ in BGC-823-shB7 group, Figure 3A–3B; t-test, *P*<0.05). Implantation of the *HOXB7*-overexpressing cell line MGC-803-B7 resulted in significantly faster tumor growth than in the MGC-803-NC group, and the tumor volumes for MGC-803-B7 (930.53 ± 129.20 mm^3^) were significantly larger than the MGC-803-NC control (186.31 ± 103.82 mm^3^) (Figure 3C–3D; t-test, *P*<0.05).

In the tail vein injection models, increased expression of *HOXB7* resulted in significantly greater numbers of macroscopic lung metastatic cancer nodules in the mouse lung tissues, with the MGC-803-B7 (100 ± 21.36) and BGC-823-shNC (112.5 ± 17.25) groups showing increased foci relative to MGC-803-NC (15.83 ± 4.84) and BGC-823-shB7 group (17.5 ± 5.88) (t-test, *P*<0.05). These results indicate that HOXB7 expression level could promote GC subcutaneous growth and lung metastases *in vivo*.

Moreover, IHC staining of the subcutaneous implantation and lung metastases of the MGC-803-B7 and BGC-823-shNC groups displayed significantly elevated Ki-67 index than those in the MGC-803-NC vector and BGC-823-shB7 groups, as well as the expected stronger HOXB7 staining (*P*<0.05, Figure 3B, 3D, 3F and 3H).

### *HOXB7* expression results in MAPK and Akt pathway activation in GC cell lines

**Figure 4:**
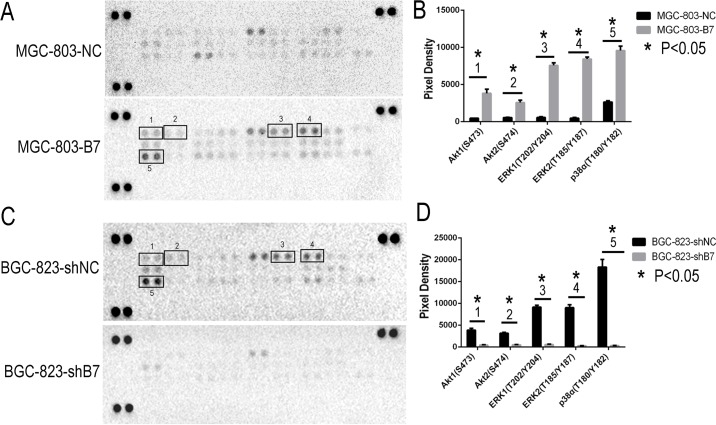
Phospho-MAPK Array Result Up-regulation the HOXB7 expression, the phosphorylation level of AKT1(S473), AKT2(S474), ERK1(T202/Y204), ERK2(T185/Y187) and p38α (T180/Y182) were significant increased in MGC-803-B7 group compared with control group, and those moleculars were remark decrease in BGC-823-shB7 group *vs.* BGC-823-shNC group. **A.** The chemiluminescence exposure pictures of Phospho-MAPK Array, between MGC-803-NC and MGC-803-B7 group. Data shown are from a 1 minute exposure to X-ray film. **B.** Five significant Pixel Density differences of Phospho-MAPK moleculars between MGC-803-NC and MGC-803-B7 group. *, *P*< 0.05. **C.** The chemiluminescence exposure pictures of Phospho-MAPK Array, between BGC-823-shNC and BGC-823-shB7 group. Data shown are from a 1 minute exposure to X-ray film. **D.** Five significant Pixel Density differences of Phospho-MAPK moleculars between BGC-823-shNC and BGC-823-shB7 group. *,*P*<0.05.

**Figure 5:**
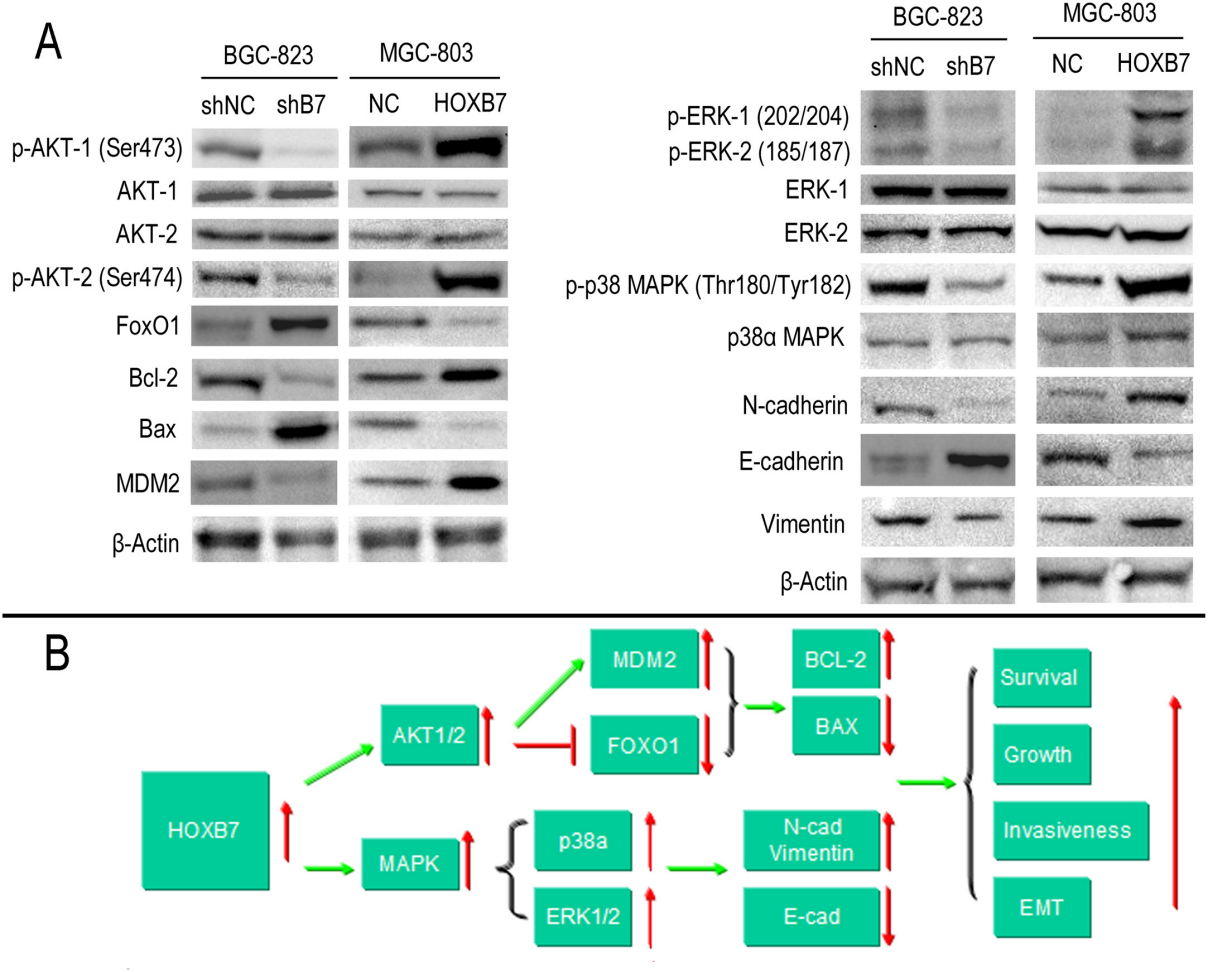
HOXB7 expression regulates the AKT-FOXO1 axis and ERK, p38α activation in gastric cancer cell lines **A.** Phosphorylation of AKT1 (S473), AKT2 (S474), ERK1 (T202/Y204), ERK2 (T185/Y187) and p38α (T180/Y182) were significantly increased in the MGC-803-B7 group compared with control group and lower in the BGC-823-shB7 group than in the BGC-823-shNC group. The expression levels of downstream targets of the AKT pathway and EMT markers, including FOXO1, BAX and E-cadherin, were down-regulated, whereas BCL-2, MDM2, N-cadherin and Vimentin were increased, in the MGC-803-B7 group compared to the MGC-803-NC group. In contrast, the opposite trend was seen in the BGC-823-shB7 compared with the corresponding negative controls. **B.** A schematic detailing the proposed model for the functional role of HOXB7 in regulating the GC cells proliferation and invasiveness.

To better understand the mechanisms that facilitate the increased cell proliferation and invasiveness seen *in vitro* and *in vivo* by expression of *HOXB7*, we looked to study differences in activation of the MAPK and AKT (downstream targets FOXO1, MDM2, BCL-2, BAX) pathways and in markers of the epithelial mesenchymal transformation (EMT, molecular markers including E-cadherin, N-cadherin, Vimentin) mediated by *HOXB7* expression. The relative levels of phosphorylation of Mitogen-Activated Protein Kinases (MAPKs) and other serine/threonine kinases were measured using the Human Phospho-MAPK Array Kit. At the same time, the downsteam members of AKT pathway and EMT markers were detected by Western blot. Phosphorylation of AKT1 (S473), AKT2 (S474), ERK1 (T202/Y204), ERK2 (T185/Y187) and p38α (T180/Y182) were significantly increased in the MGC-803-B7 group compared with control group and lower in the BGC-823-shB7 group than in the BGC-823-shNC group (Figure 4A, 5A). But the phosphorylation level of other members of MAPKs pathway in this Human Phospho-MAPK Array Kit, such as TOR(S2448), RSK2(S386), MSK2(S360), MKK6(S207/T211), MKK3(S218/T222), JNK1(T183/Y185), JNK2(T183/Y185), p53(S46), JNK2(T221/Y223), HSP27(S78/S82), GSK-3β(S9), GSK-3α/β(S21/S9), p38β(T180/Y182), p38δ(T180/Y182), p38γ(T180/Y182) and CREB(S133) were not affected by HOXB7 expression (Figure 4). Furthermore, in the MGC-803-B7 group compared to the empty vector control cell line (MGC-803-NC), the expression levels of downstream targets of the AKT pathway and EMT markers, including FOXO1, BAX and E-cadherin, were down-regulated, whereas BCL-2, MDM2, N-cadherin and Vimentin were increased (Figure 5A). In contrast, the opposite trends were seen in the BGC-823-shB7 group, compared with the corresponding negative controls (Figure 5A). FOXO1 plays a pivotal role in tumor suppression by inducing growth arrest and apoptosis, and is an important target in AKT signaling pathways. AKT negatively regulates FOXO1 by direct phosphorylation, resulting in its inactivation and sequestration into the cytoplasm [18]. In this study, we showed that AKT could be activated by *HOXB7* overexpression in GC cell lines, so we further sought to determine whether this difference in FOXO1 expression was mediated by a difference subcellular localization by immunofluorescence. The cells overexpressing *HOXB7* (MGC-803-B7) demonstrate more cytoplasmic localization of FOXO1 than the negative control MGC-803-NC cells. In contrast, more nucleus localization of FOXO1 was observed in HOXB7 knock-down cell (BGC-823-shB7) than the negative control BGC-823-shNC cells (Supplementary Figure S1). These data indicate that a cytoplasmic subcellular location of FOXO1 was associated with increased HOXB7 expression.

Because tumor epithelial mesenchymal transformation plays an important role in tumor invasiveness, a series of EMT markers were also examined by Western blot. When HOXB7 was overexpressed in MGC-803 cells, the epithelial marker E-cadherin was decreased and the mesenchymal markers N-cadherin and vimentin were increased. When the expression of HOXB7 in BGC-823 was knocked down by HOXB7-shRNA, the opposite trend in expression was observed (Figure 5A). Additionally, the MGC-803-B7 line overexpressing *HOXB7* displayed a more elongated, spindle cellular morphology, which may be consistent with the increased EMT markers observed (Supplemental Figure S1).

These findings indicate that HOXB7 likely promotes GC cells proliferation and survival in part through the AKT1/2-FOXO1-BCL-2-BAX axis. Two major family members of the MAPK pathway, ERK1/2 and p38α, which were increased in the context of *HOXB7* overexpression, may play an important role in GC invasiveness and spread, in part by promoting the cancer cell epithelial mesenchymal transformation (Figure 5B).

In order to further investigate the role of HOXB7 and its effect on AKT/MAPK signaling and cell migration and proliferation, three commercially available kinase inhibitors (p38 inhibitor IV, FR180204, Akt1/2 inhibitor) were selected to block AKT and MAPK signaling. Addition of these inhibitors significantly reduced the invasion and proliferation ability of both MGC-803-B7 and control MGC-803 cell lines both in the presence and absence of HOXB7 expression. However, treatment with these AKT/MAPK inhibitors resulted in less inhibitory effects on cell invasion and proliferation in the GC lines overexpressing HOXB7 (Supplementary Figure S2). These data indicate that the AKT/MAPK signaling pathway accounts for at least part of the *HOXB7*-mediated increase in invasiveness and proliferation, and that targeting this axis can inhibit these features in GC cell lines.

### *HOXB7* expression is increased in GC patient tissue and associated with poor prognosis

**Figure 6:**
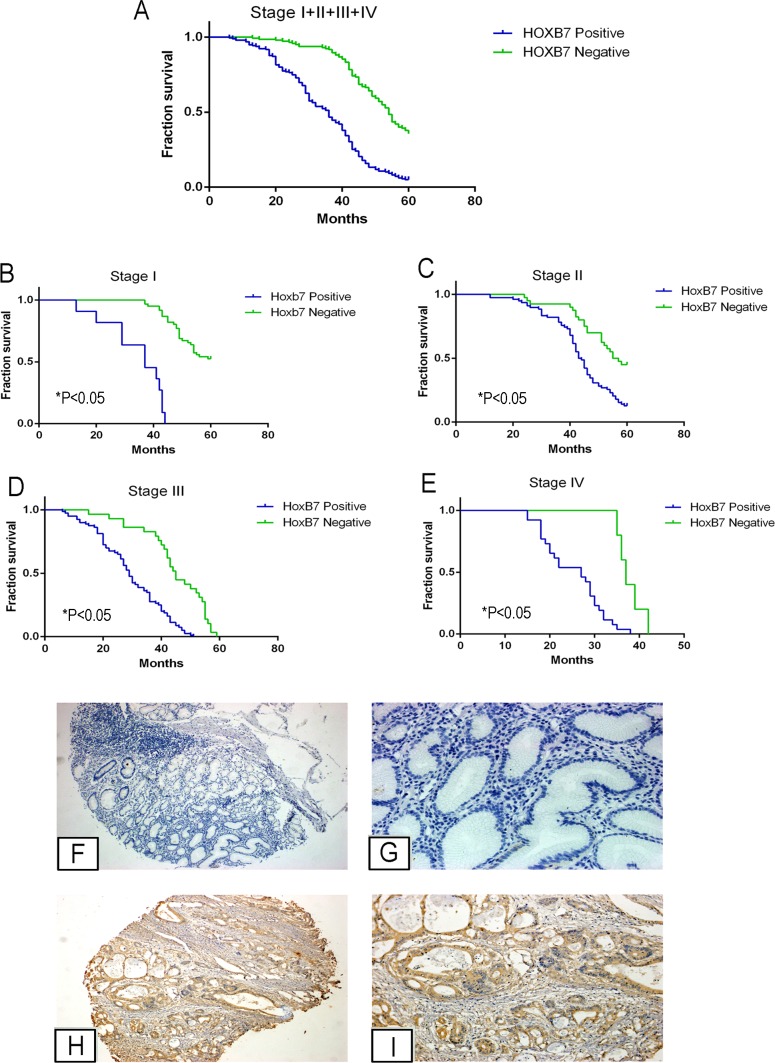
HOXB7 expression in gastric cancer patient tissues correlates with decreased overall survival and more aggressive tumor characteristics **A-E.** Kaplan-Meier analysis of survival curves in GC patients. Survival was assessed by Kaplan-Meier analysis in (A) all GC patient tissues (n=330) and in (B) Stage I, (C) Stage II, (D) Stage III, (E) Stage IV (each Stage comparison and in overall group, P<0.05). **F-I.** Representative IHC images of HOXB7 expression in normal gastric epithelium and GC specimens. No HOXB7 staining was detected in normal gastric epithelium (F, G), whereas it was positively detected in GC tissues (H, I). G and I, magnification at 200×, were magnified portions of Figures F and H, respectively.

To further study the relationship between HOXB7 expression, clinical pathological parameters and prognosis, we first sought to validate the trend of increased *HOXB7* expression in gastric tumor tissue. We used immunohistochemical staining for HOXB7 in a GC tissue microarray (TMA) containing 330 patient samples (Table 1). Based on the HOXB7 immunoreactivity scores, elevated levels of HOXB7 were detected in 59.1% (195/330) of cases, and decreased levels of HOXB7 were detected in 40.9% (135/330) of cases (Figure 6F–6I). HOXB7 staining was confined to cancerous portions of tissues examined (Supplementary Figure S3).

**Table 1:** Association between HOXB7 expression and clinicopathological factors

Clinical parameters	HOXB7			
	Low	High	t/*χ*^2^	*P*
Age(yrs)	56.55±11.20	58.72±10.73	3.161	0.076
Gender			0.212	0.645
Male	98(41.7%)	137(58.3%)		
Female	37(38.9%)	58(61.1%)		
Location			1.892	0.388
Distal	71(42.0%)	98(58.0%)		
Middle	52(42.6%)	70(57.4%)		
Proximal	12(30.8%)	27(69.2%)		
Size			15.644	0.000
≥5cm	34(27.2%)	91(72.8%)		
<5cm	101(49.3%)	104(50.7%)		
Histology type			2.435	0.487
Papillary adenocarcinoma	5(35.7%)	9(64.3%)		
Tubular adenocarcinoma	97(39.3%)	150(60.7%)		
Mucinous adenocarcinoma	9(40.9%)	13(59.1%)		
Signet-ring cell carcinoma	24(51.1%)	23(48.9%)		
Lauren classification			56.834	0.000
Diffuse type	104(60.5%)	68(39.5%)		
Intestinal type	31(19.6%)	127(80.4%)		
Differentiation			3.595	0.309
Well	1(63.6%)	6(36.4%)		
Moderately	38(41.9%)	62(58.1%)		
Poorly	96(34.0%)	126(66.0%)		
Undifferentiation	0(33.3%)	1(66.7%)		
Invasion Depth (T Grade)			52.823	0.000
T1	37(84.1%)	7(15.9%)		
T2	35(53.8%)	30(46.2%)		
T3	59(29.1%)	144(70.9%)		
T4	4(22.2%)	14(77.8%)		
Lymphatic Metastasis (N Grade)			55.579	0.000
N0	79(68.1%)	37(31.9%)		
N1	18(29.0%)	44(71.0%)		
N2	19(28.4%)	48(71.6%)		
N3	19(22.4%)	66(77.6%)		
Distant metastasis (M Grade)			8.691	0.003
M0	130(43.5%)	169(56.5%)		
M1	5(16.1%)	26(83.9%)		
TNM Stages			76.673	0.000
I	61(84.7%)	11(15.3%)		
II	40(33.9%)	78(66.1%)		
III	29(26.6%)	80(73.4%)		
IV	5(16.1%)	26(83.9%)		
Lymphatic metastasis			54.724	0.000
Yes	56(26.2%)	158(73.8%)		
No	79(68.9%)	37(31.9%)		
Vascular invasion			48.071	0.000
No	87(63.0%)	51(25.0%)		
Yes	48(37.0%)	144(75.0%)		

All cases were classified according to the World Health Organization's (2010) pathological classification of gastric cancer. Invasion Depth (T Grade) grade T1 includes T1a and T1b, T4 includes T4a and T4b. Lymphatic Metastasis (N Grade) grade N3 includes N3a and N3b. TNM grade I includes Ia and Ib, TNM grade II includes IIa and IIb, TNM grade III includes IIIa, IIIb and IIIc.

The HOXB7 positive staining rate in the patients with lymph node metastasis was greater (73.8%, or 158/214) than in patients without lymph node metastasis (31.9%, or 37/116, *P*<0.05). Additionally, the HOXB7 positive rate in patients with distant metastasis (83.9%, or 26/31) was also higher than that in patients without distant metastasis (56.5%, or 169/299; *P*<0.05). HOXB7 positivity was also correlated with tumor size, as 72.8% of tumors from GC patients (size ≥5cm) showed positive HOXB7 staining, compared with 50.7% of GC tumors < 5cm (*P*<0.05). HOXB7 positive staining was also correlated with tumor grade, as 83.9% of Grade IV tumors showed HOXB7 staining while only 15.3% of Grade I GC tumors were positive (*P*<0.05). Finally, higher levels of tumors bearing HOXB7 positive-staining was also found to be correlated with Lauren classification, invasion depth (T Grade), and lymphatic metastasis (N Grade) (*P*<0.05, Table 1), but were not associated with age, sex, location, and histological type of GC (*P*>0.05, Table 1).

We also analyzed the relationship between HOXB7 expression and the prognosis of GC patients. Based on HOXB7 staining of tumors from this cohort of GC patients (n= 330), the mean survival time in patients with HOXB7 tumors was significantly shorter than that of the HOXB7 negative tumors (34. 83 ± 13.26 months *vs*. 50.91 ± 10.10 months, respectively, *P*<0.05), as was the 5-year survival rate (5.1% *vs*. 37.0%, *P*<0.05, Figure 6A), which was consistent across each TNM stage (Figure 6B–6E, *P*<0.05). Cox multivariate analysis showed that TNM stage, Lauren classification, Vascular invasion and HOXB7 expression were independent prognostic factors (Table 2).

**Table 2:** Multivariates analysis as determined by Cox regression analysis in 330 GC patients

Clinicopathological Parameters	95% Confidential Interval		Hazard Ratio	*P* value
	Lower	Upper		
Lauren classification	1.248	2.467	10.452	0.001
Vascular invasion	0.204	0.983	4.006	0.045
TNM stage	1.044	2.345	4.695	0.030
HOXB7 expression	2.267	4.389	46.471	0.000

## DISCUSSION

Previous studies have demonstrated that cancers often exhibit aberrant expression of genes with functions in embryogenesis, particularly the Homeobox group. Expression of homeobox genes can induce tissue growth that contributes to tumor onset and progression [19]. HOXB7, a member of the HOX family of homeodomain transcription factors, is a critical developmental regulator of cancer cells. Increased expression of *HOXB7* has been reported in several malignancies and has been implicated in influencing a number of cellular processes, including cell invasion, DNA repair, metastasis, and angiogenesis, and is thought to contribute to tumorigenesis and poor survival in many cancers [6–10, 15, 20]. In this study, we studied the effect of HOXB7 on cell proliferation and invasiveness by both *in vitro* and *in vivo* experiments with GC cell lines, and also investigated the prognostic significance of *HOXB7* expression in a larger series of GC patient samples. In GC patients, increased expression of HOXB7 was observed in cancer tissue compared with adjacent normal tissues. Increased expression of *HOXB7 in GC cell* lines significantly increased the proliferation, colony formation and migration ability of GC cells *in vitro*. Our *in vivo* results show that over-expression of *HOXB7* significantly accelerates subcutaneous tumor growth and increases the number of lung metastases. In contrast, these promotion effects were reversed by decreased expression of HOXB7 expression in GC cell lines (mediated by HOXB7-targeting shRNA), which was observed both *in vitro* and *in vivo*. Furthermore, in a cohort of 330 patient GC tumors, our study showed that HOXB7 immuno-positivity was observed in 59.1% of GC patients and was associated with increased tumor size, depth of invasion, lymph node metastasis, distant metastasis, and TNM stage. Moreover, the patients with positive HOXB7 staining had shorter survival time and poorer prognosis across all tumor stages.

AKT is a primary signaling transducer of the phosphoinositide 3-kinase (PI3K) pathway and contributes to cancer progression by inhibiting apoptosis, promoting cell proliferation and regulating migration and invasion in many cancer types [21–22]. Previous studies have shown that the PI3K/AKT pathway is frequently activated in GC and revealed that PI3K/AKT signal transduction pathway participates in cell proliferation with regulators such as FOXO1, BCL-2 and BAX [23–24]. Sasaki et al [22] reported that activation of the AKT pathway in GC could promote cancer cell proliferation and mediate cancer cell migration. Our results indicate that increased the expression of HOXB7 promoted the proliferation and invasion ability of GC cell lines and that these lines exhibited increased AKT1 (S473) and AKT2 (S474) phosphorylation. Furthermore, the downstream AKT pathway molecules associated with cell apoptosis and survival, such as FOXO1, BCL-2 and BAX, were also involved in facilitating this effect. We therefore speculate that the HOXB7-mediated increase in GC cell line proliferation and invasiveness is at least partly dependent on effects on the AKT signaling pathway

The MAPKs signaling pathway, including P38, ERK1/2 and JNK1/2, plays a crucial role in tumor cell proliferation, adhesion and migration [25–31] and these pathways are known to be activated in GC [32–33]. Epithelial to mesenchymal transition plays an important role in cancer invasion and metastasis and is characterized by a reduction in expression of epithelial markers, such as E-cadherin, while other mesenchymal markers and transcription factors, such as N-cadherin, snail, slug, and, vimentin, are up-regulated [34]. Previous studies showed that MAPKs signaling pathway was involved in EMT procession [35–36], include the ERK, p38 or JNK kinase pathway. Herein, we examined the effect of *HOXB7* expression on MAPKs pathway-associated key functional protein kinases in GC cell lines, using the Human Phospho-MAPK Array Kit and Western blotting assays, as well as the effect on key EMT associated proteins. We found that ERK1 (T202/Y204), ERK2 (T185/Y187) and p38α (T180/Y182) phosphorylation were involved in the dynamic expression of HOXB7 in GC cell lines, while our data indicate that in this context the JNK pathway members, such as JNK1 (T183/Y185), JNK2 (T183/Y185), JNK2 (T221/Y223), are likely not involved. Additionally, we found that HOXB7 expression increased the expression of the key functional proteins of EMT, such as N-cadherin and vimentin, and these cell lines also exhibited increased invasion and proliferation. Therefore, HOXB7 enhancement of the migration and invasion ability of GC cells may be mediated by ERK and p38α pathways, two members of MAPKs pathway, at the phosphorylation level, resulting in a series of enhanced invasive phenotype changes, including EMT. However, the mechanism how HOXB7 affects AKT or MAPK signaling and whether HOXB7 could directly regulate the upstream molecules of them, such as PTEN, PI3K, MKK3/6 and RTKs, still need to be further explored.

In conclusion, our findings suggest that increased expression of *HOXB7* might be a valuable prognostic marker of GC progression through its potential role in promoting tumorigenesis, invasiveness and disease spread. Thus, modulation of tumor proliferation and invasiveness through inhibiting the activation of AKT or MAPKs (ERK and p38α) mediated by *HOXB7* expression may be a promising therapeutic target for GC prevention and therapy.

## MATERIALS AND METHODS

### Cell culture

Human GC cell lines MKN-45, MKN-28, SGC-7901, BGC-823, HGC-27, AGS and human gastric mucosal epithelial cell line GES-1 were purchased from the Cell Bank of Shanghai Institute of Cell Biology (Shanghai, China). They were cultured in RPMI-1640 culture media (HyClone, USA) with 10% Fetal Bovine Serum (FBS) and maintained at 37°C in 5% CO_2_ and passaged at 80-90% confluency every three or four days.

### Vector construction and transfection

The HOXB7 construct was generated by subcloning PCR-amplified full-length human HOXB7 cDNA into pcDNA3.1 for overexpression. siRNA (GCCCTCTTTAATGCTGTCTTT) was employed for downregulation. The HOXB7-shRNA hairpin DNA sequence was annealed and synthesized as: Forward, CACC GCCCTCTTTAATGCTGTCTTTCTCGAGAAAGACAGCATTAAAGAGGGCTTTTTTG and Reverse: AGCTCAAAAAAGCCCTCTTTAATGCTGTCTTTCTCGAGAAAGACAGCATTAAAGAGGGC. It was cloned into pYr-1.1 vector (Yinrun Biotechnology, Changsha, China) and linearized by restriction enzymes BsaI. Lipofectamine 2000 (Invitrogen, USA) was used for transfection according to the manufacturer's protocol. Stable cell lines expressing HOXB7 (MGC-803-B7) or shHOXB7 (BGC-823-shB7) were selected after 3-4 weeks by treating 200 mg/mL G418. Cells transfected with pcDNA3.1 or the pYr-1.1 vector (containing non-targeting control shRNA sequence) were considered as negative controls and named as MGC-803-Vector and BGC-823-shNC, respectively.

### RNA extraction and qRT-PCR

RNA was extracted and purified from cell or tissue homogenates by using Trizol method. cDNA was prepared using Superscript cDNA synthesis kit (TAKARA, Japan) following the manufacturer's protocol. qRT-PCR was carried out using SYBR Premix Ex Taq (Takara, Japan) with cDNA-specific primers. GAPDH functioned as internal control and relative expression level was calculated using the 2^−ΔΔ^CT method. The mRNA specific primers for qRT-PCR as follows: HOXB7-F: GTCCCTGCCTACAAATCATC and HOXB7-R: GAAG CAAACG CACAAGAAGT; GAPDH-F: TGAAGGT CGGAGTCAACGG and GAPDH-R: CTGGAAGATG GTGATGGGATT. The PCR parameters were as follows: 95°C for 4 min, followed by 40 cycles of 95°C for 10 s, 58°C for 30 s and 72°C for 30 s. At the end of the PCR cycles, melting curve analysis was performed.

### MTs assay

Cellular proliferation was assayed using The Cell Proliferation MTs Assay Kit (G3580, Promega, Japan) following the manufacturer's protocol. All cell lines (stable over-expression and down-regulation of HOXB7 cells and their corresponding negative control cells) were seeded into 96-well plates with 3×10^3^ cells in 200 μL culture medium per well. After attachment, 20μL of MTs reagents were added to each well every 24 hours. After incubating for an additional 4 hours, the absorbance was measured at 490 nm.

### Colony formation assays

All cell lines (stable over-expression and down-regulation of HOXB7 cells and their corresponding negative control cells) were seeded in 6-well plates with 100 cells and a final volume of 2 mL culture medium per well. The cells were maintained at 37°C in 5% CO_2_ and the culture medium was changed every four days for the following three weeks. At the end of the experiment, the colony formation cells of each group were stained with H&E staining.

### Migration and invasion assays

The cell migration and invasion assays were done in 24-well Transwell plates with or without pre-coated Matrigel, as described previously [37]. For migration assay, the stable cells and the corresponding negative control cells were seeded in the upper chamber of the Transwell system (8.0 mm, pore size; 3422, Millipore, USA) with 1×10^4^ cells/well in 100 μL of serum-free RPMI-1640 medium, and the lower chamber was filled with 600μL 30% FBS RPMI-1640 culture medium. For invasion assay, 2×10^5^ cells/well of the stable cells and the corresponding negative control cells were seeded in the upper chamber with pre-coated Matrigel (8.0 mm, pore size; ECM554, Millipore, USA), and the lower chamber was filled with 600 μL 30% FBS RPMI-1640 culture medium. After 24h or 48h of incubation, cells remaining on the top layers of the inserts were removed by cotton swab scrubbing, and cells on the lower surface of the membrane were fixed and stained with H&E staining. The cell numbers in five random fields (200×) were counted for each chamber, and the average value was calculated.

### Phospho-MAPK array detection and western-blotting

The Human Phospho-MAPK Array Kit (ARY002B, R&D Systems, USA) was employed to detect the relative levels of phosphorylation of 26 kinases of all three major families of mitogen activated protein kinases (MAPKs), the extracellular signal-regulated kinases (ERK1/2), c-Jun N-terminal kinases (JNK1-3), and different p38 isoforms (α/β/δ/γ) to understand the effect of HOBX7 on these signaling pathways.

Cells were collected and lysed in cell lysates buffer and pipetted up and down to re-suspend and rock the lysates gently at 2-8°C for 30 min, centrifuged at 14000g for 5 min, and the supernatant was transferred into a clean test tube. Sample protein concentrations were quantitated using the BCA method and then the extractions were aliquoted and stored at ≤-80°C.

The Array Detection steps were followed according to the manufacturer's protocol. Array membranes were blocked by using Array Blocking Buffer for one hour. A total of 200μg protein of each sample were added to separate tubes and adjust to a final volume of 1.5 mL with Array Buffer 1, and then 20 μL of Detection Antibody Cocktail was added to every separate tubes and incubated at room temperature for one hour. After blocking the membranes, the prepared Sample/Detection Antibody Cocktail Antibody mixtures were added into the 4-Well Multi-dish and incubated overnight at 2-8°C. After washing the membranes for three times, the diluted Streptavidin-HRP buffer was added and incubated for 30 minutes. After washing three times, the membranes were incubated with Chemi Reagent Mix and theChemiluminescence signal was detected using the Chemiluminescence Gel Imaging System (Bio-Rad, USA).

For Western blotting, cells were washed in phosphate-buffered saline and lysed in protein lysis buffer (1% NP-40, 20 mmol/l Tris–HCl (pH 8), 137 mmol/l NaCl, 10% glycerol, 2 mmol/l EDTA). 40 μg of total protein was used for Western Blotting along with rabbit polyclonal antibodies against p44 MAP Kinase (Erk1) (#4372, 1:1000, CST, Cell Signaling, USA), p42 MAP Kinase (Erk2) (#9108, 1:1000, Cell Signaling, USA), Erk1 (pT202/pY204), Erk2 (pT185/pY187) (ab136926, 1:2000, ABCAM, USA), AKT-1 (#2938, 1:2000, CST, Cell Signaling, USA), Akt2 (#2964, 1:2000, CST, Cell Signaling, USA) Phospho-Akt2 (Ser474) (#8599, 1:2000, CST, Cell Signaling, USA), Phospho-Akt1 (Ser473) (#9018, 1:1000, CST, Cell Signaling, USA), Phospho-p38 MAPK (Thr180/Tyr182) (#9211, 1:2000, CST, Cell Signaling, USA), Bcl-2 (D55G8) (#4223, 1:3000, CST, Cell Signaling, USA), Bax (D2E11) (#5023, 1:1000, CST, Cell Signaling, USA), Vimentin (D21H3) (#5741, 1:5000, CST, Cell Signaling, USA), E-Cadherin (24E10) (#3195, 1:1000, CST, Cell Signaling, USA), FoxO1 (C29H4) (#2880, 1:3000, CST, Cell Signaling, USA), HOXB7 polyclonal antibody (H00003217-D01P, 1:400, Abnova, Taiwan) and p38α MAPK (L53F8) Mouse mAb (#9228, 1:1000, CST, Cell Signaling, USA). Blots were probed with antibodies against β-Actin (D6A8) (#8457, 1:5000, CST, Cell Signaling, USA) as an internal control.

### Immunofluorescence

In order to localize FOXO1 in GC cell immunofluorescence assay was performed on cell lines. GC cells, such as BGC-823-shNC, BGC-823-shB7, MGC-803-NC and MGC-803-B7, were washed briefly in 1x PBS and fixed in 4% paraformaldehyde for 15 min at 37°C, respectively. Cells were then permeabilized by inncubating with 0.5% Triton X for 15 min at room temperature. After washing, cells were incubated in blocking buffer (5% of BSA) for 30 min at 37°C. All the cell lines were incubated with anti-human FOXO1 rabbit polyclonal antibody (#2880; 1:1000, CST, Cell Signaling, USA) overnight at 4°C. After washing, cells were incubated with Cy3-labeled anti-rabbit antibody (#A0521, 1:1000, Beyotime Biotechnology, China) for 30 min at room temperature, and nuclei were then counter-stained with DAPI (#C1005, Beyotime Biotechnology, China).

### AKT/MAPK signaling validation

In order to validate whether HOXB7 truly regulates the migration and proliferation process via AKT/MAPK signaling, **t**hree commercially available inhibitors, Akt1/2 kinase inhibitor(A6730, SIGMA, USA), FR180204 (ERK inhibitor II, SML0320, SIGMA, USA) and p38 MAP Kinase Inhibitor IV (SML0543, SIGMA, USA), were choosenfor blocking AKT, ERK and p38α kinase activations, respectively, according the manufacturer's instructions. After MGC-803 cells adhered to the six-well plate, 200 nM of Akt1/2 kinase inhibitor, 200 nM of FR180204 and 130 nM of p38 MAP Kinase Inhibitor IV, were added into the well for blocking the corresponding kinase activation, respectively. After 24h later, the MGC-803 cells were transfected with HOXB7 plasmid, to increase the expression of HOXB7, followed by collected for doing invasion and proliferation assays.

### Xenograft analysis

Four to five-week-old female BALB/c athymic nude mice were purchased from Slac Laboratory Animal Co. Ltd. (Shanghai, China). All mice were housed and maintained under specific pathogen-free conditions and used in accordance with institutional guidelines and approved by the Use Committee for Animal Care.

To evaluate the effect of HOXB7 on gastric tumor progression, mice were inoculated subcutaneously in the right flank with a total of 2 × 10^6^ cells which were suspended in 100 μL PBS, of MGC-803-B7, BGC-823-shB7, MGC-803-Vector or BGC-823-shNC cells. Tumor size was measured by a slide caliper and tumor volume was calculated as (length×width^2^)/2 every week.

To evaluate the effect of HOXB7 on gastric tumor metastasis, a total of 1×10^6^ cells of BGC-823-shNC, BGC-823-shB7, MGC-803-Vector or MGC-803-B7 cells were injected into the lateral tail vein. Metastatic lung nodules were quantified after H&E staining using a dissecting microscope at endpoint.

After mice were sacrificed, subcutaneous tumor tissues or Lung metastastic tissues were rapidly taken out, a part of the tissue was put into liquid nitrogen frozen immediately and the rest of it was fixed in 10% neutral buffered formalin and embedded in paraffin. Sections of 4 μm were cut and stained with H&E and IHC staining using antibodies of HOXB7 and Ki-67 (#GA62661, Clone MIB-1, Ready to use, DAKO, USA). The Ki-67 proliferation index was determined by counting 1000 cells in hot spots and calculated as the percentage of positive nuclei by one senior pathologist.

### Patient samples and tumor tissue microarray (TMA)

Thirty-six paired GC and adjacent non-tumor tissues were obtained from patients with primary gastric adenocarcinoma without any history of radiation or chemotherapy treatment prior to surgery were obtained at Zhejiang Provincial People's Hospital, Hangzhou, Zhejiang Province, China. After surgical removal, the cancer tissues were frozen immediately in liquid nitrogen and stored until extraction of RNA and protein.

In addition, 330 cases of GC samples were collected from gastrointestinal surgery and the department of pathology of Zhejiang Provincial People's Hospital, from January 1998 to January 2004. All of the patients have been followed up for over 5 years with the deadline of December 2009. The survival time was counted from the date of surgery to the follow-up deadline or date of death, which was mostly caused by carcinoma recurrence or metastasis. No patients received any radiotherapy or chemotherapy prior to surgery, and written informed consent was obtained before analysis. The GC patients' age ranged from 17 to 80 (with median as 58.0 years old) and all cases were classified according to the World Health Organization's pathological classification (2010) of tumors. The clinicopathological characteristics of the GC patients are summarized in Table 1.

The core tumor area (tumor occupying >50%) of every GC tissue wax block was determined and labeled by the pathologist through H&E stained sections. Then, every 35 cases of the core cancer tissues (about 2mm diameter) were taken from individual paraffin embedded GC blocks and were arranged in recipient paraffin microarray blocks (tissue array blocks) using a trephine. Finally 10 tissue array blocks were made, containing a total of 330 cases of the GC samples described above. Each block contained more than three internal controls consisting of normal gastric mucosa.

### IHC staining and evaluation

The GC patient tumor tissue microarray (TMA) as described above was used for HOXB7 immunohistochemical detection. Each 4 μm TMA section was deparaffinized, rehydrated and then rinsed with PBS. Antigen retrieval was carried out in 0.01 M citrate buffer (pH 6.0) for 3 min using high pressure retrieval method. Then the sections were incubated with 3% H_2_O_2_ for 10 min followed by 10% normal goat serum for 15 min at room temperature, in order to block endogenous peroxidase and non-specific antigen. Then the sections were incubated with rabbit anti-human HOXB7 polyclonal antibody (1:400 dilutions in PBS, H00003217-D01P, Abnova, Taiwan) overnight at 4°C. After rinsing with PBS for three times, the sections were incubated with biotin labeled secondary antibody for 20 min at room temperature, and then incubated with horseradish peroxidase conjugate polymer (Invitrogen, USA) for another 20 min at room temperature. Finally, 3, 3-diaminobenzidine (DAB) was used to visualize the signal development, and then the sections were counterstained with hematoxylin.

The immunoreactivity levels of each case were estimated under a light microscope by assessing the average signal intensity (on a scale of 0–3) and the proportion of cells showing positive staining (0, <5%; 1, 5–25%; 2, 26–50%; 3, 51–75%; 4, 76–100%) and were independently carried out by two pathologists without knowledge of the clinical data, as described previously [38]. The intensity and proportion scores were then multiplied to obtain a composite score; 0–3 was defined as negative and 4–12 as positive.

### Statistical analysis

All statistical analyses were performed using the SPSS 13.0 statistical software. Comparisons between groups were performed with a 2-tailed paired Student's t test. The relationships between HOXB7 expression and clinicopathologic characteristics were tested using the Chi-square test. Survival curves were plotted by Kaplan-Meier method and compared by log-rank test. The significance of various survival related variables was assessed by Cox regression model in the multivariate analysis. *P*<0.05 was considered statistically significant.

## SUPPLEMENTARY FIGURES


