# Supplementary material for: HOXB7 overexpression promotes cell proliferation and correlates with poor prognosis in gastric cancer patients by inducing expression of both AKT and MARKs
Source: Oncotarget. 2016 Nov 25;8(1):1247–61. doi: 10.18632/oncotarget.13604 (PMC5352052; doi:10.18632/oncotarget.13604)
Supplement: Supplementary file 1 [file oncotarget-08-1247-s001.pdf]

HOXB7 overexpression promotes cell proliferation and correlates with poor prognosis in gastric cancer patients by inducing expression of both AKT and MARKs

SUPPLEMENTARY FIGURES

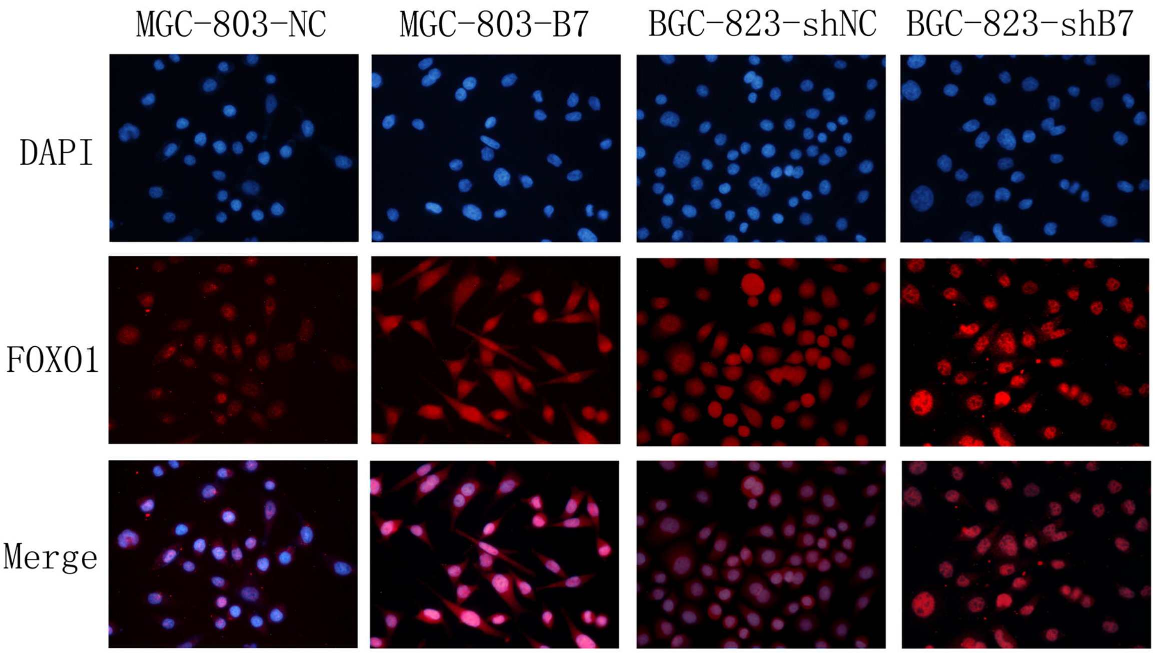

Supplementary Figure S1: FOXO1 Immunofluorescence shows differences in subcellular localization in different HOXB7 expression GC cells. The cells that overexpress *HOXB7* (MGC-803-B7) have long spindle shapes which may be an indication of the cells undergoing epithelial mesenchymal transformation. Additionally, these cell lines demonstrate more cytoplasmic subcellular localization of FOXO1 than the negative control MGC-803-NC cells. In contrast, FOXO1 exhibited a nuclear localization in the cell line with HOXB7 knockdown (BGC-823-shB7) when compared to the negative control BGC-823-shNC cell line.

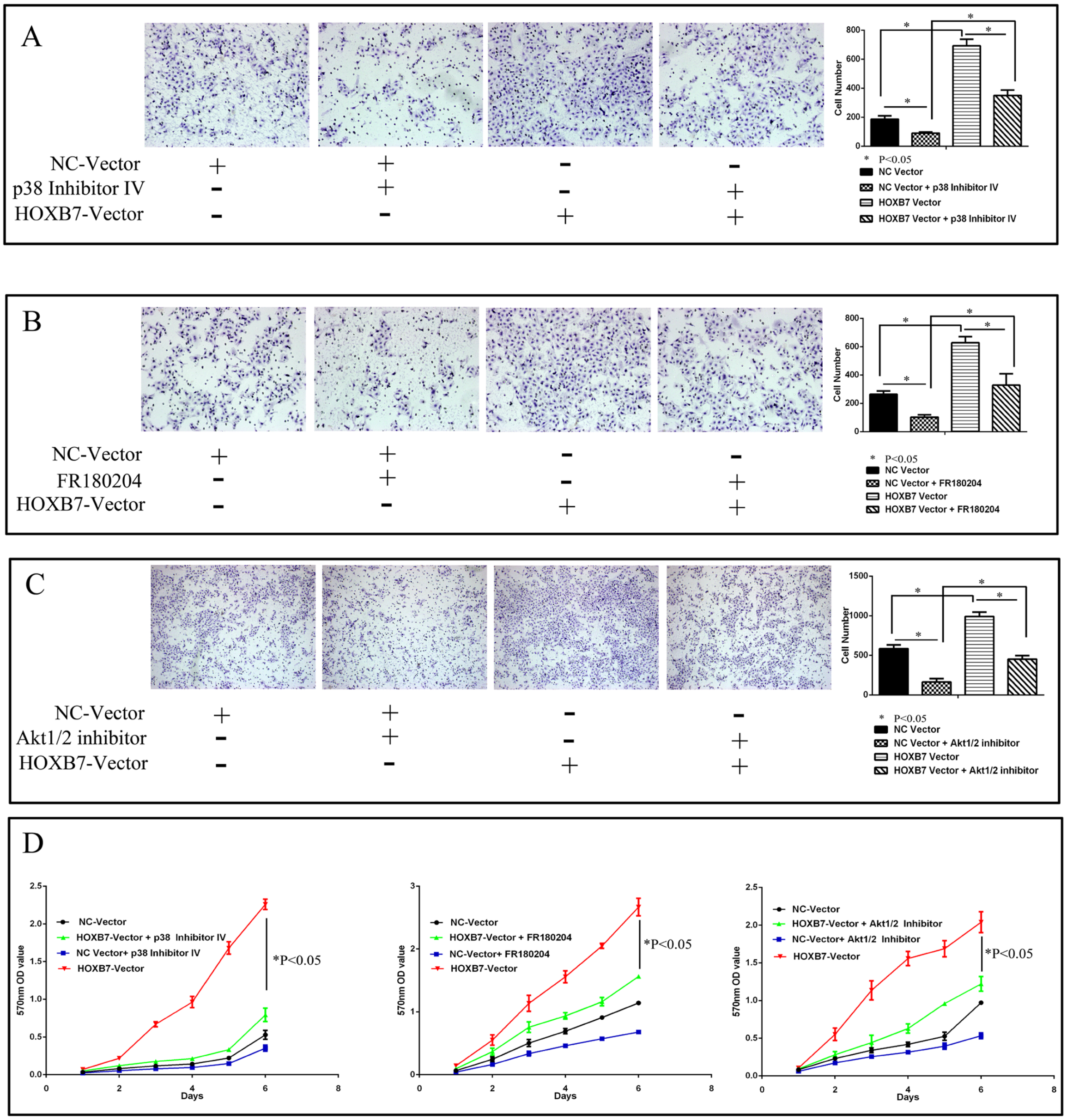

Supplementary Figure S2: Inhibition of the AKT/MAPK signaling pathways using compounds results in decreased gastric cancer cell line invasion and proliferation. AKT/MAPK inhibitors decrease the invasiveness of MGC-803 cells, after treated with the **A.** p38α signal inhibitor IV, **B.** ERK inhibitor FR180204 and **C.** AKT1/2 inhibitor, in the context of HOXB7 overexpression or the vector group (*P<0.05). **D.** Treatment with AKT/MAPK inhibitors also significantly decreases MGC-803 cell proliferation, with HOXB7 expressing cell lines showing significantly increased proliferation compared to the treated lines and NC-vector group (*P<0.05).

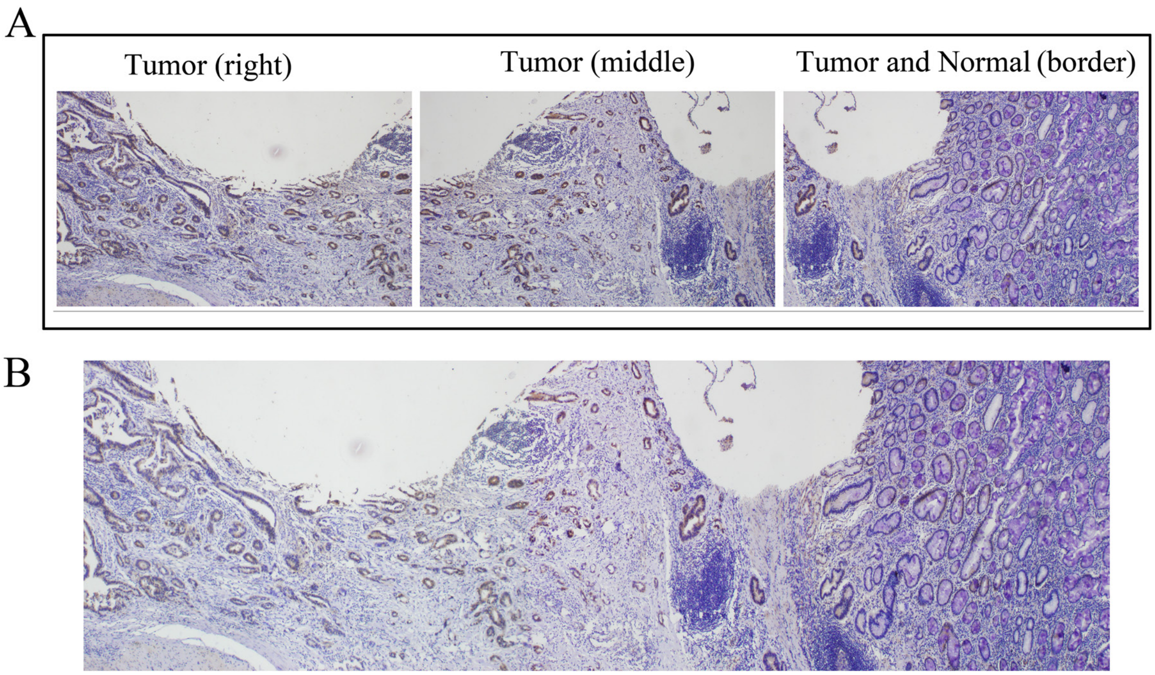

Supplementary Figure S3: HOXB7 expression in a patient GC tissue shows tumor-specific HOXB7 staining. A. Three continuously and different regions in GC tissue section, from tumor side to the adjacent normal side. **B.** The merged image of these three figures. HOXB7 staining was negative in adjacent normal tissue and positive in GC cancer cells. The defect area in the picture serves as a donate tissue for making TMA blocks.
